# Root traits contributing to plant productivity under drought

**DOI:** 10.3389/fpls.2013.00442

**Published:** 2013-11-05

**Authors:** Louise H. Comas, Steven R. Becker, Von Mark V. Cruz, Patrick F. Byrne, David A. Dierig

**Affiliations:** ^1^Water Management Research, United States Department of Agriculture-Agricultural Research ServiceFort Collins, CO, USA; ^2^Department of Soil and Crop Sciences, Colorado State UniversityFort Collins, CO, USA; ^3^National Center for Genetic Resources Preservation, United States Department of Agriculture-Agricultural Research ServiceFort Collins, CO, USA; ^4^Bioagricultural Sciences and Pest Management, Colorado State UniversityFort Collins, CO, USA

**Keywords:** root morphology, root architecture, hydraulic conductance, hydraulic conductivity, QTL, drought tolerance, MAS

## Abstract

Geneticists and breeders are positioned to breed plants with root traits that improve productivity under drought. However, a better understanding of root functional traits and how traits are related to whole plant strategies to increase crop productivity under different drought conditions is needed. Root traits associated with maintaining plant productivity under drought include small fine root diameters, long specific root length, and considerable root length density, especially at depths in soil with available water. In environments with late season water deficits, small xylem diameters in targeted seminal roots save soil water deep in the soil profile for use during crop maturation and result in improved yields. Capacity for deep root growth and large xylem diameters in deep roots may also improve root acquisition of water when ample water at depth is available. Xylem pit anatomy that makes xylem less “leaky” and prone to cavitation warrants further exploration holding promise that such traits may improve plant productivity in water-limited environments without negatively impacting yield under adequate water conditions. Rapid resumption of root growth following soil rewetting may improve plant productivity under episodic drought. Genetic control of many of these traits through breeding appears feasible. Several recent reviews have covered methods for screening root traits but an appreciation for the complexity of root systems (e.g., functional differences between fine and coarse roots) needs to be paired with these methods to successfully identify relevant traits for crop improvement. Screening of root traits at early stages in plant development can proxy traits at mature stages but verification is needed on a case by case basis that traits are linked to increased crop productivity under drought. Examples in lesquerella (*Physaria*) and rice (*Oryza*) show approaches to phenotyping of root traits and current understanding of root trait genetics for breeding.

## INTRODUCTION

Water shortages are responsible for the greatest crop losses around the world and are expected to worsen, heightening international interest in crop drought tolerance. Within the U.S. alone, about 67% of crop losses over the last 50 years have been due to drought. The 2012 drought in the U.S. was the worst in 60 years and more frequent occurrences of water shortages are expected due to climate projections and increasing competition for water among urban, industrial, and agricultural demand (IPCC, 2012; Haro von Mogel, 2013). Geneticists and breeders are in position to make strides in breeding plants for better yields under drought. Drought tolerance is most desirable as the maintenance of crop productivity under drought (definition of drought tolerance in this paper; Passioura, 2007), which can be accomplished in a variety of ways, including drought avoidance or desiccation prevention, potentially in combination, through matching crop water use with water availability, and recovery of growth following rewetting (Passioura, 2012). While the shoot drives water uptake through a plant, root system size, properties, and distribution ultimately determine plant access to water, and thus, set limits on shoot functioning, similar to an analogy of a horse driving a cart and the cart setting limits on the capacity of the horse (Nardini et al., 2002; Sperry et al., 2002). Thus, an area of recent interest is improvements of root traits that increase efficient deployment of tissues for foraging of soil water and, expressly, the maintenance of productivity under water deficit. However, key questions remain: *which root traits help most and under what conditions?*

**FIGURE 1:**
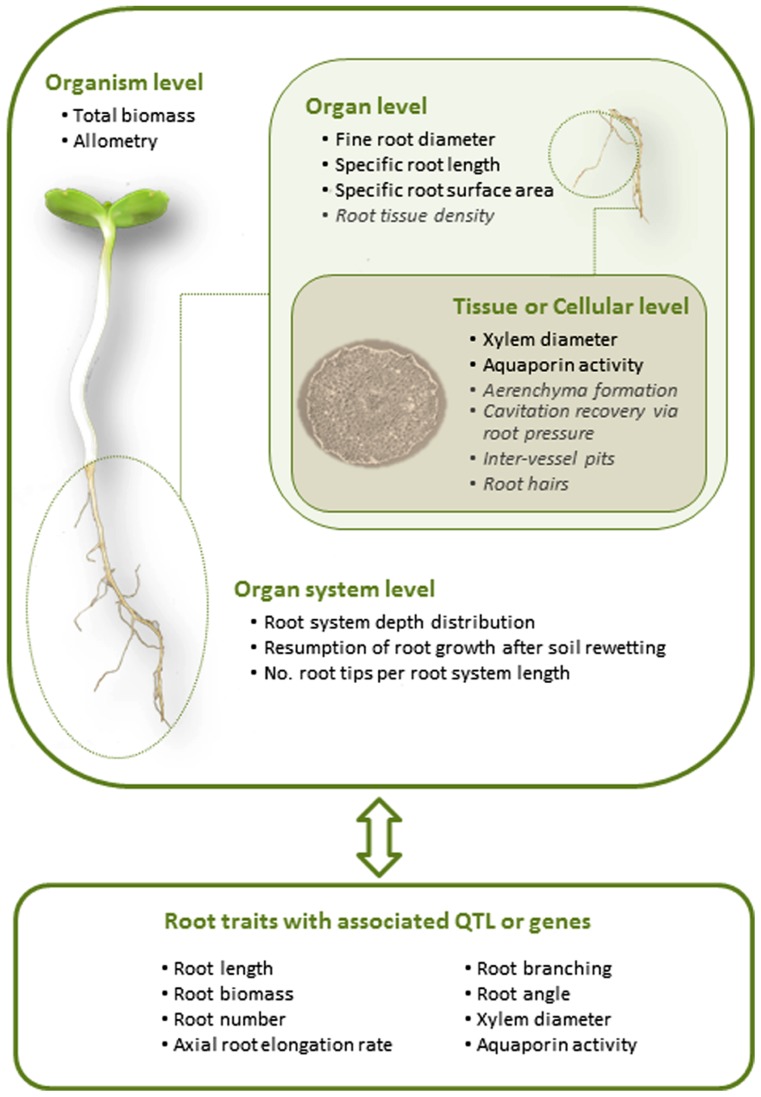
**Areas of focus of plant studies seeking to understand root traits related to plant productivity under water shortages and genetic screening of traits to identify their coding.** Organism level refers to whole plant traits, organ system to the entire root system (fine and coarse roots, as the shoot system would refer to leaves and stems), organ to single root types (e.g., fine roots), and tissue or cell to single cell types (e.g., xylem or cortical cells). Root traits in black text are traits that have been shown to be related to drought tolerance, and gray italic those that may be associated with drought tolerance but either require more research or have been equivocal.

Past efforts in improvement of germplasm for water-limited environments have been accomplished by focusing on specific traits for particular crops and drought conditions, which appear more clearly when viewed through a framework that dissects the benchmark of water-limited yield potential into independent components (Passioura and Angus, 2010). An appreciation of the growth strategies of individual crops and specifics of particular drought conditions crops face will need to continue to be at the forefront of successful breeding programs. In agricultural systems without irrigation (dryland systems), drought may be episodic in varying degrees or extend through the majority of the growing season. These different scenarios of drought will have different impacts on crop growth and development above and below ground (Passioura, 2012). In irrigated agriculture, water may be applied in varying degrees of deficit irrigation throughout the season, as full irrigation during strategic periods of the season, or applied in different combinations of deficit and full irrigation during different periods of the growing season. Breeding efforts will also be more successful if coupled to advances being made in management (Kirkegaard and Hunt, 2010). It is widely recognized that breeding efforts need to account for the genotype by environment by management (G × E × M) interaction because improving crop productivity will require breeding for different plant traits and growth strategies in different environments and under different management (Sinclair et al., 2010; Passioura, 2012; Reynolds et al., 2012). Nevertheless, a few generalizations in root traits associated with crop productivity under drought are beginning to emerge (Wasson et al., 2012). Discussion of these root traits and others resulting from advances in the plant ecophysiological arena are the subject of this review and will be discussed at the organism, organ system, organ, and tissue and cellular level (**Figure 1**).

## ROOT SYSTEMS, TRAITS, AND FUNCTIONING IN WATER UPTAKE

Before considering specific root traits, it is worth discussing root systems as a whole. There is a level of complexity in root systems of both woody and herbaceous plants that is crucial to root system functions but often goes unacknowledged: the root system is not one organ but rather composed of two, and sometimes three, main types of root organs. For woody plants, coarse woody roots, mirroring stems aboveground, serve functions of perennial structures, anchorage, carbohydrate and nutrient storage during the season, and transport of nutrients and water. The fine roots of woody plants, which are limited to the terminal two root segments (referred to as first and second branch orders counting back from root tips), serve ephemeral roles in foraging for belowground resources (Guo et al., 2008; Xia et al., 2010). The root system of herbaceous plants, crop and non-crop alike, is also comprised of coarse and fine roots, which may correspond to tap versus lateral roots in a tap root system or seminal and nodal versus lateral roots in a fibrous root system (Fitter, 2002). Like in woody plants, coarse and fine roots of herbaceous plants can be distinguished by a jump in diameter class, which tends to occur between the terminal two root orders and the rest of the root system. Coarse roots of herbaceous plants serve functions of anchorage and typically establish overall root system architecture, controlling ultimate rooting depth, and the ability of plants to grow into compacted soil layers (e.g., Henry et al., 2011). In addition to coarse seminal roots, nodal roots (or brace roots in maize, *Zea mays*) developing from lower portions of the stem provide additional opportunities for plant foraging of late-season precipitation with different responses to soil water than the primary root system (Rostamza et al., 2013). Finally, fine (or lateral) roots are the most active portion of the root system in water uptake, and comprise the majority of the length and surface area of these root systems in herbaceous and woody plants alike (Bauhus and Messier, 1999; Rewald et al., 2011).

### ROOT SYSTEM SIZE AND ALLOMETRY

The size of a plant’s root system is a key trait of interest related to acquisition of soil resources but only when considered in relation to the size of the remainder of the plant, either relative to leaf area, shoot, or whole plant size. Shifts in allometry (metrics of root to shoot relationships) and shoot stature can compensate for water shortage, and, along with shifts in stand densities, can maintain stomatal conductance under xeric conditions similar to levels under mesic conditions (Mencuccini, 2003; Addington et al., 2006; Maseda and Fernandez, 2006 and references within). Allometry is typically measured as root:shoot ratio of dry mass. When determined from biomass, root biomass per total plant biomass (i.e., root mass fraction, RMF) is a more robust quantification of the relative size of root systems for statistical reasons but has been less frequently used (Reich, 2002). Ultimately, ratios of root to leaf surface area (*A*_R_:*A*_L_) or root length:leaf area ratio are more functionally descriptive than mass fractions of tissues and can be used as a surrogate for water uptake capacity in proportion to capacity for light interception, as well as providing the surface area of water uptake versus transpiration loss (e.g., Sperry et al., 2002; Diaz-Espejo et al., 2012).

Functional equilibrium theory suggests that plants shift allocation among absorptive tissues to acquire resources that most limit growth (Brouwer, 1983). Optimal partitioning theory takes this idea one step further and suggests that plants allocate resources among organs to optimize whole plant growth (Thornley, 1969; Bloom et al., 1985). These theories suggest plants may be adapted to produce a particular root:shoot ratio but this ratio will shift to balance resources limiting growth with a degree of plasticity, or responsiveness, which is a trait of interest in and of itself (Shipley and Meziane, 2002; but see Reynolds and D’Antonio, 1996). Root:shoot ratio changes with plant growth and development in addition to shifting in response to limiting resources above versus below ground. Therefore, care must be taken to control for plant size and ontology, especially when assessed on young plants (Müller et al., 2000). When ratios of dry mass fractions (e.g., root:shoot ratio; RMF) are taken instead of *A*_R_:*A*_L_, these ratios may be too coarse of a measure to be meaningful in many cases (Reynolds and D’Antonio, 1996 and references within). Ratios of dry mass fractions do not account for the more plastic response of tissue morphology, architecture, and physiology (e.g., Boot and Mensink, 1990; Jackson et al., 1990; Aerts et al., 1991; Van der Vijver et al., 1993; Berntson et al., 1995; Ryser and Lambers, 1995). This is crucial because root dry mass fractions can mask shifts in root morphology or architecture by remaining constant while the total length or surface area of a root system increases or decreases dramatically with relatively small shifts in root diameter, specific root length (SRL; root length per dry mass), specific surface area (SSA; root surface area per dry mass), or proportion of coarse to fine roots.

### ORGAN, TISSUE, AND CELLULAR LEVEL TRAITS

At the organ level, several root morphological traits for both fine and coarse portions of root systems have been found to be associated with increased productivity under drought. Key morphological traits seem to be traits that influence total root length and surface area of root systems and include root diameter, root tissue density, SRL, and SSA (Fitter, 2002; Nardini et al., 2002). Root diameter and tissue density control the length and surface area of root systems for a given biomass allocated to the root system (Fitter, 2002), which not only controls the amount of surface directly interacting between roots and soil, but also the amount of root surface colonized by mycorrhizal fungi assisting in plant nutrient acquisition (Smith and Read, 2008). SRL and SSA summarize the overall effect of both root diameter and tissue density in terms of root length per dry biomass invested in the tissue (Fitter, 2002). For woody plants, root diameter predominately controls differences in SRL and SSA among species, with root tissue density affecting plasticity within species due to plant responses to edaphic factors such as soil water (Comas et al., 2002; Comas and Eissenstat, 2009). Small diameter roots with greater SRL enable plants to efficiently increase hydraulic conductance by increasing surface area in contact with soil water, increasing the volume of soil that can be explored for water, and, also, increasing root hydraulic conductivity by decreasing the apoplastic barrier of water entering the xylem (Eissenstat and Achor, 1999; Rieger and Litvin, 1999; Huang and Eissenstat, 2000; Solari et al., 2006; Hernández et al., 2010; Comas et al., 2012). Accordingly, decrease in root diameter has been proposed as a trait for increasing plant acquisition of water and productivity under drought (Wasson et al., 2012). In addition to root morphological traits affecting water and nutrient acquisition through control of root length and surface area, root morphology also affects resource acquisition by influencing root growth rate, with finer roots associated with faster root growth rate (Eissenstat, 1991; Robinson et al., 1991, 1999). Both woody and herbaceous plants adapted to dry conditions are found to have smaller diameter fine roots with greater SRL (Hernández et al., 2010; Henry et al., 2012).

A few additional root attributes have been associated with increased productivity under drought. Root tissue density was found to primarily control differences in SRL and SSA among several grass species (Ryser and Lambers, 1995; Wahl and Ryser, 2000). Aerenchyma formation in the root cortex can decrease root tissue density, increasing SRL and SSA (Zhu et al., 2010). Induction of root aerenchyma has been proposed to increase plant performance and improve carbon economy under drought in maize (Zhu et al., 2010). However, aerenchyma impeded radial movement of water through the root cortex and decreased water uptake in water-stressed rice (Yang et al., 2012a). Root hairs produced in many species can also substantially increase root surface area and are particularly responsive to reductions in soil water and nutrient availability (Bhat et al., 1979; Claassen and Jungk, 1982; Mackay and Barber, 1985; Bates and Lynch, 2001), although benefits under low soil water may not be found for all plants or conditions (Wen and Schnable, 1994; Suzuki et al., 2003). Root hairs in rice, for example, were found to be more important for nutrient uptake and provided no significant impact on water uptake (Suzuki et al., 2003). However, increases in root surface area *via* root hairs may compensate for reductions in root elongation occurring in extremely dry soils (Mackay and Barber, 1985). Root hairs may also promote root contact with soil particles as soil dries and may thus assist roots in acquiring soil water (Wasson et al., 2012 and references within). Additionally, increased abundance and conductance of aquaporins, which regulate the passage of water uptake, may increase root hydraulic conductivity (conductance per length of root) to meet shoot demand and compensate for reduced root surface area (e.g., Kaldenhoff et al., 1998; Parent et al., 2009; Vandeleur et al., 2009; Laur and Hacke, 2013).

New root tips, and, thus, continual root growth to produce these tips, may be more important for the uptake of mobile resources than the total amount of root length and surface area (Robinson et al., 1991). The main zones of water uptake are young root tips (Sanderson, 1983; Haussling et al., 1988; Peterson et al., 1993; Varney et al., 1993; Kramer and Boyer, 1995). Although, even for mobile soil resources, total root length and surface area may matter when plants compete (Newman and Andrews, 1973). Roots increase apoplastic barriers and take up less water with age and exposure to dry soil (Steudle, 2000), which may appear unfavorable. However, models show greater water uptake for the same amount of root length when a small proportion of the root system is unsuberized (e.g., when only root tips are unsuberized) because there is greater hydraulic conductance along the root axis, in contrast to that of a “leaky pipe” (Zwieniecki et al., 2003).

In addition to root diameter, xylem diameter also affects root hydraulic conductivity and can affect plant productivity under drought (Zimmermann, 1983; Tyree et al., 1994). Research to some degree supports generalizations that plants with large diameter xylem vessels have greater hydraulic conductivity, but less conservative water use and greater risk of cavitation than those with small diameter vessels (Richards and Passioura, 1989; Sperry and Saliendra, 1994; Tyree et al., 1994; Alder et al., 1996; Gallardo et al., 1996) but exceptions can be found (Pockman and Sperry, 2000). Cavitation and embolism formation set thresholds on stomatal closure, with safety margins needed varying with frequency and amount of drought that plants are adapted to handle (Choat et al., 2012). As a breeding strategy, a general reduction in root xylem diameter can reduce total plant hydraulic conductance under well-watered conditions and limit plant maximum growth potential, therefore, when breeding these traits, programs have targeted their expression specifically in roots that function in water uptake primarily under dry conditions (Passioura, 1983). An Australian wheat (*Triticum aestivum*) breeding program successfully developed wheat varieties with more conservative hydraulic architecture in seminal roots to save soil water under drought for critical stages in crop yield development later in the field season (Passioura, 1972; Richards and Passioura, 1989). In this case, a general decrease in root hydraulic conductance was not manifested under well-watered conditions when seminal roots played a minor role and nodal roots predominately acquired water for the plant (Richards and Passioura, 1989).

Exceptional species with large diameter xylem adapted to dry environments have been found (Pockman and Sperry, 2000). These species are able to maintain high transpiration rates and conductivity but have high resistance to cavitation (Smith et al., 1996; Pockman and Sperry, 2000). Identifying mechanisms in such examples may be of special interest to breeding programs because such mechanisms would avoid reduced maximum yield potential under favorable growing condition. Mechanisms at work in such examples may be related to the anatomy of intervessel pit areas and greater rarity of “leaky” pits, which minimizes the initiation of cavitation (Wheeler et al., 2005; Christman et al., 2009).

### ROOT SYSTEM GROWTH AND DISTRIBUTION UNDER DROUGHT: NUANCES RELATED TO FIELD CONDITIONS AND GENOTYPES

Of all root traits of potential importance, plant allometry and hydraulic conductance during drought have been of keen interest and the subject of several reviews (Mencuccini, 2003; Maseda and Fernandez, 2006; Wasson et al., 2012). Although shifts in root growth and allometry may increase plant hydraulic conductance and productivity under drought (Mencuccini, 2003; Addington et al., 2006; Maseda and Fernandez, 2006), plant allometric responses partially depend on soil properties and spatio-temporal formation of drought. The “balanced growth” hypothesis (sensu Bloom et al., 1985) suggests that some plants respond to drought by stimulating or maintaining root growth while reducing shoot growth. Increased root versus shoot growth should improve plant hydraulic status under mild or moderate drought stress due to increased root to leaf surface, continued production of new root tips, and enhancement of plant capacity for acquiring water to support existing shoots. The underlying mechanisms behind the shift in allometry are difference in the sensitivity of root and shoot growth to water stress (Hsiao and Xu, 2000). Even partial drying of root systems can lead to decreased allocation to vegetative shoots (e.g., Dry et al., 2001). It has been observed, however, that under severe water deficits, limited root growth may occur because of very low soil water availability and high soil impedance (Taylor and Gardner, 1963; Cornish et al., 1984; van Zyl, 1984; Comas et al., 2005). In this case, as mentioned in the previous section, increased root hair and aquaporin production may play particularly important roles in compensating for reductions in root elongation and surface area production.

**FIGURE 2:**
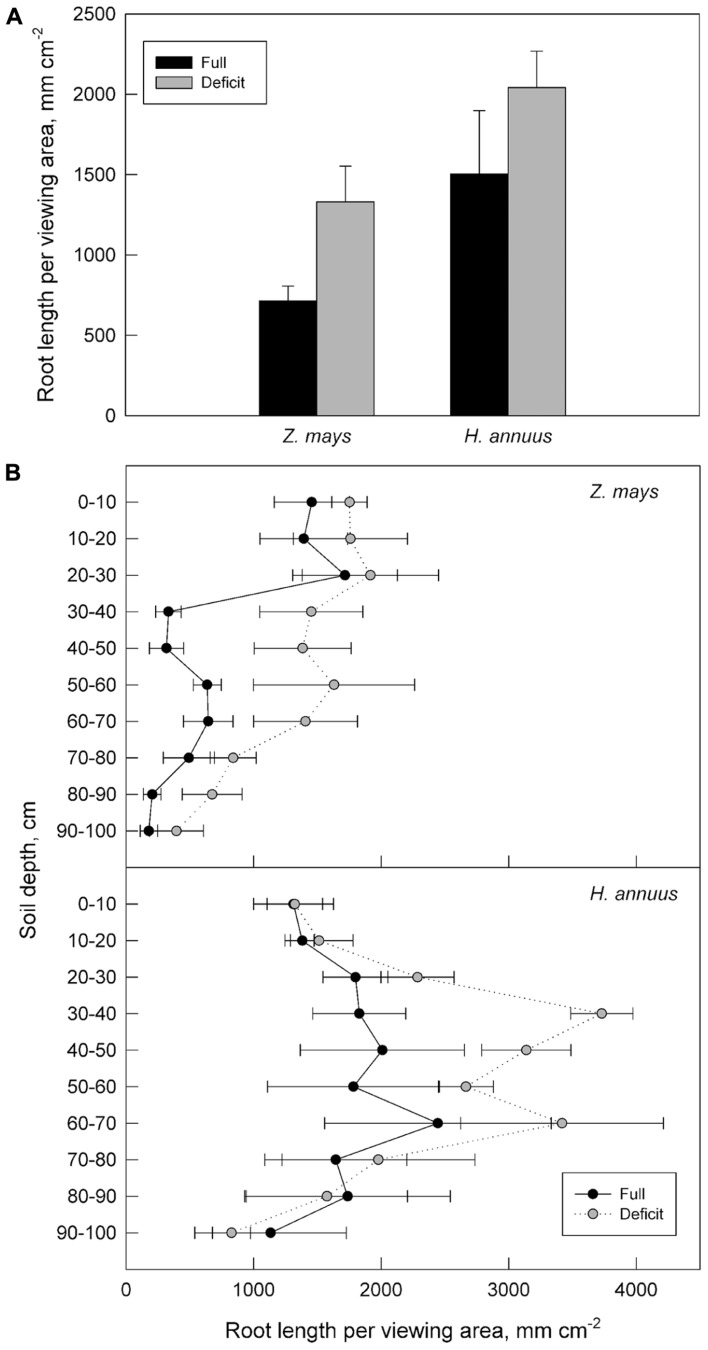
**The production of root length and its distribution for fully and deficit irrigated maize and sunflower over the 2012 growing season in minirhizotron windows at the USDA-ARS Limited Irrigation Research Farm in Greeley, CO, USA (40.45°, -104.64°, 1430 m).** Root growth is expressed in terms of root length per viewing area of minirhizotron window for two crops contrasting in hydraulic strategies grown under full and deficit irrigation. Total annual root growth in viewing windows down to 100 cm **(A)** as well as in 10 cm increments of soil depth **(B)** are given. Each bar and point represents root growth averaged among four minirhizotron tubes per treatment, with each tube installed in a different treatment plot. Soil at the site is a sandy loam. Annual precipitation is approximately 350 mm. Irrigation is applied with pressure-compensated surface drip. Deficit irrigation is applied to achieve a targeted 40% of full evapotranspiration (ET) irrigated treatment during deficit periods in late vegetative and maturation growth phases (V7-V21 and R3-R6 in maize; V8-R2 and R6-R9 in sunflower).

Additionally, the ability of plants to grow roots according to distribution of available soil water profoundly increases plant productivity under drought. Root traits for water acquisition from deep in the soil profile and methods of such trait assessment have been well described in recent reviews (Wasson et al., 2012). Plants are inherently somewhat plastic in their root distribution, especially deep-rooted species such as maize and sunflower (*Helianthus annuus*; **Figure 2**). Irrigation reached to approximately 30 cm soil depth in the crops illustrated but roots of these crops were found below 1 m. Deep roots for water acquisition deep in the soil profile may be especially important for smaller statured plants, such as wheat, rice, and common bean (*Phaseolus vulgaris*), but have generally conferred advantages for plants growing under limited soil water in agricultural and natural systems (Ho et al., 2005; Schenk and Jackson, 2005; Hund et al., 2009; Lopes and Reynolds, 2010; Henry et al., 2011; but see Sun et al., 2011). As soil dries at the surface, water may be available deeper in the profile than many agricultural species are adapted to reach, and require root system development deeper in the profile to access this water. In this case, breeding for plants with less root length density (RLD, root length per soil volume) in shallow soil layers and increased RLD in medium and deep layers has been proposed as an efficient growth strategy in environments where deep water could be available to crops later in the growing season (Wasson et al., 2012; Lynch, 2013). In addition to root distribution, root architecture that includes greater hierarchical structure may promote hydraulic lift and allow for greater utilization of water available deep in the soil profile (Doussan et al., 2006). In cases where deep water availability could promote crop productivity directly or *via* hydraulic redistribution, larger diameter xylem vessels may be advantageous to increase axial hydraulic conductivity of roots growing in deeper soil layers (Wasson et al., 2012). Transpiration supplied by hydraulic lift or redistribution may be large enough to support plants through extreme drought episodes even if the total amount of water redistributed is small.

Where drought is episodic, plant response to rewetting of soil is equally important for maintenance of yield under drought as water extraction and hydraulic functioning in drying soil (Sperry et al., 2002). In many woody plants, hydraulic failure occurs in roots rather than shoots because xylem in roots is more prone to cavitation than in shoots (Pockman and Sperry, 2000 and references within). Structural impediments to water uptake in root systems that develop under stress may require regrowth of roots with plant recovery contingent on this regrowth (Lo Gullo et al., 1998). Recovery through new root growth may be species specific, as demonstrated by examples of evergreen tree species unable to repair extensive loss of root hydraulic conductance to resume water uptake (Hacke et al., 2000), whereas drought-adapted genotypes of wheat respond rapidly to rewetting by producing “rain roots,” similar to desert succulents (North and Nobel, 1991; Sadras and Rodriguez, 2007). Where drought is episodic but perhaps less severe, nocturnal refilling of embolized xylem *via* root pressure appears to play an critical role for resumption of hydraulic conductance in herbaceous crops, potentially providing an important additional area for breeders to improve drought tolerance (Sperry et al., 2003; Stiller et al., 2003, 2005; Sperry, 2011).

**FIGURE 3:**
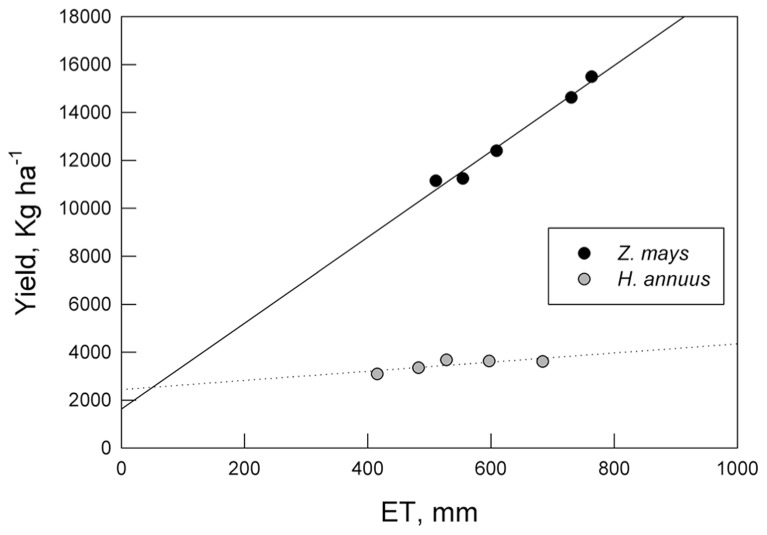
**Crop yield per harvest area and crop evapotranspiration (ET) for the same study shown in Figure 2**.

**FIGURE 4:**
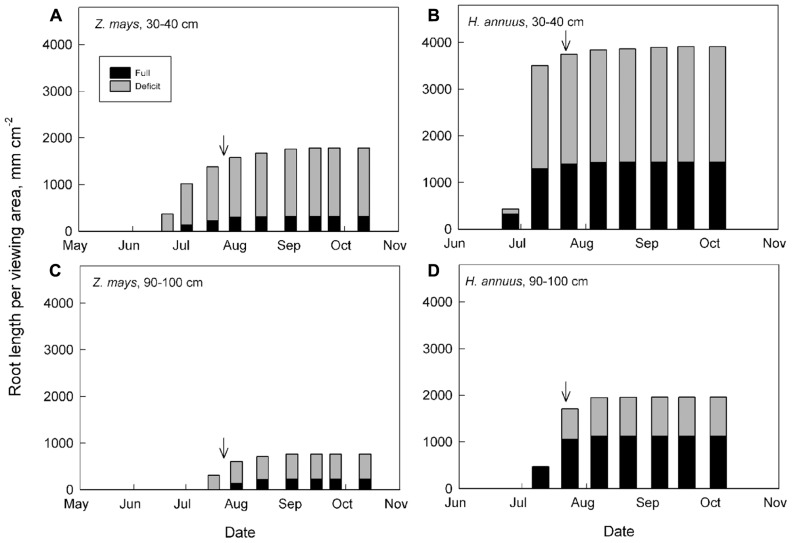
**Seasonal root growth of fully and deficit irrigated maize and sunflower in two soil depths.** Root growth across the season at two soil depths for *Z. mays* **(A,C)** and *H. annuus* **(B,D)** is from the same study shown in **Figure 2.** Each bar represents root growth averaged among four minirhizotron tubes per treatment. Arrows indicate the beginning of the critical reproductive phase for each crop (R1 in maize, July 23; R3 in sunflower, July 20).

Root allocation and distribution may depend on plant growth strategies and their general response to water deficits and distribution of available soil water. Maize has high water use efficiency (WUE) but is sensitive to water shortages (**Figure 3**; Ghannoum, 2009). Maize, which has more conservative hydraulic conductance compared to sunflower, decreases transpiration more quickly than sunflower, which maintains carbon assimilation during drought, even during the course of wilting (Comas, personal observation). Both maize and sunflower decrease shoot size, and increase *A*_R_:*A*_L_ and relative root distribution to deeper depths in response to water deficits, although sunflower, emblematic of a drought avoider, has a generally deeper root system than maize and redistributes an even greater percentage of its roots to deeper soil depths (**Figure 2**). Root growth in both maize and sunflower continues longer into the season than shoot vegetative growth and the onset of reproduction, with the capacity for even greater overlap of root growth with reproduction under water deficit (**Figure 4**). As breeding for plant productivity under drought advances, it may be advantageous to consider whole plant strategies and root traits and patterns of spatio-temporal growth with a systems approach. Working with two crops with contrasting hydraulic responses, we might expect different traits to improve productivity under drought in these crops, which highlights the need to take specifics of the genotype, as well as environment and management, into account.

## GENETICS OF ROOT TRAITS UNDER DROUGHT

### CHALLENGES IN UNDERSTANDING AND UTILIZING GENETIC ANALYSES OF ROOT TRAITS

Most root traits are controlled by multiple genes, each governing small effects and often with a degree of epistasis or interaction effects that can change with environmental conditions (de Dorlodot et al., 2007; Cooper et al., 2009). The quantitative trait loci (QTL) that contribute to root traits can be considered either constitutive or adaptive, the classification of which can be useful in selecting traits most beneficial in the target environment (Collins et al., 2008).

Both adaptive and constitutive root traits can be difficult to phenotype. Therefore, it is not surprising that a majority of genetic research has focused on above-ground traits while the “hidden half” of the plant is much less represented in recent research (Herder et al., 2010). A search for rice (*Oryza sativa* L.) QTL associated with drought in the database TropGene (Hamelin et al., 2013) revealed 139 QTL in only five studies for root traits under drought stress, while non-root traits consisted of 387 QTL in 15 studies. A common approach to phenotyping for genetic research is the use of controlled growing environments such as greenhouse pots or tubes, growth chambers, hydroponic systems, and agar gel. However, caution must be used when applying these procedures to root morphology studies, as frequent inconsistencies of QTL and gene locations are often caused by a lack in quality and quantity of phenotypic information (Collins et al., 2008; Xu and Crouch, 2008; Hargreaves et al., 2009; Wojciechowski et al., 2009). In a maize study for gene expression under drought, Barker et al. (2005) reported that 27% of gene expression was up- or down-regulated when stressed for 5 days in buckets as compared to only 5% differential regulation when plants were stressed over 5 weeks in the field. The same study also reported that genes regulated in buckets tended to differ from those regulated in field conditions. These differences may be related to the involvement of different mechanisms in short-, medium-, and long-term response to drought (Maseda and Fernandez, 2006). To the extent that differences among studies are related to environmental differences, the compilation of these studies could lead to the identification of constitutive gene and trait expressions that are crucial across multiple environments for improving drought tolerance in the field.

Traits such as rooting depth and RLD in wheat and chickpea (*Cicer arietinum*), respectively, have shown high heritability across different environments and have also been related to improvements in grain yield under certain conditions (Kashiwagi et al., 2005; Sayar et al., 2007). Phenotypic and genotypic variation for highly heritable traits such as these in controlled environments is more likely to be similar to variation under field conditions. However, cases where phenotypes at mature developmental stages were highly responsive to soil and climatic conditions, and showed different results from those in controlled conditions emphasize the need for thorough field validation (Watt et al., 2013).

### GENES AND QTL ASSOCIATED WITH ROOT TRAITS UNDER DROUGHT

A number of studies have reported QTL linked to traits associated with increasing the foraging capacity of root systems. These include in rice: increased root length (Price et al., 2002; MacMillan et al., 2006; Courtois et al., 2009), root biomass (Courtois et al., 2003), and root number (Zheng et al., 2000, 2003; Courtois et al., 2009); in wheat: increased total root biomass, length and number of roots (Sharma et al., 2011), seminal root angle and number (Christopher et al., 2013; but see Giuliani et al., 2005b for contrasting strategy in maize), and deep root growth and seminal root number (Hamada et al., 2012); and in maize: increased root number, branching, dry mass, and decreased diameter and root angle (Giuliani et al., 2005b), and lateral and axial root length, and axial root elongation rate (Ruta et al., 2010). Increased root biomass, RLD and rooting depth are often considered to be primary drivers of drought avoidance (Kashiwagi et al., 2005). It is also possible that these traits are associated with stable QTL that are expressed in multiple environments. In a meta-QTL analysis, Courtois et al. (2009) identified 119 root QTL in rice from 24 studies. Many of these QTL, primarily for maximum root length, were associated with “hot spots” on chromosomes 1 and 9, which contained QTL detected in multiple populations and environments.

In addition to QTL, some specific genes or mechanisms have been associated with variation for root traits in major cereal crops. Reduced height and semi-dwarfing genes are common in many modern wheat (Evans, 1998) and barley (*Hordeum vulgare*) varieties (Chloupek et al., 2006). Semi-dwarfing genes of barley have been shown to contribute to greater root system size (measured by electrical capacitance) than non-semi-dwarf alleles at the same loci (Chloupek et al., 2006). However, Wojciechowski et al. (2009) found inconsistent effects of these genes for root length and root architecture traits in different types of growing media.

Genotypic variation or plasticity in deep rooting capacity in rice has been associated with productivity under drought stress (Kato et al., 2006; MacMillan et al., 2006; Steele et al., 2006). Increased water uptake associated with greater deep root length and SRL was linked to a large-effect QTL in rice that also contributes to improvements in yield under severe drought stress (Bernier et al., 2009). More recently Uga et al. (2013) have identified and cloned the *DRO1* gene in rice on chromosome 9 which is associated with rooting depth due to an increased gravitropic response in root tips. After backcross introgression of this gene into the IR64 variety of rice an increase in drought tolerance was seen in drought environments with no apparent reduction in grain yield under well-watered conditions.

In maize, a major constitutive QTL, designated *Root-ABA1*, was associated with crown root branching, diameter, and angle, as well as whole root dry mass (Giuliani et al., 2005b). Being a constitutive QTL, it was detected consistently across different water regimes in both greenhouse and field settings. In the model plant *Arabidopsis thaliana*, researchers have identified QTL for ABA induced reduction in lateral root growth as well as root system plasticity and size (Fitz Gerald et al., 2006; Xiong et al., 2006). Finally, increases in water uptake have also been associated with the up-regulation of aquaporin genes *PIP1* and *RWC-3* in maize, which shows that root physiology, in additional to or concurrent with shifts in root system size, can be associated with increased capacity of root systems to acquire water (Giuliani et al., 2005a).

### MARKER ASSISTED SELECTION AND INTROGRESSION IN CEREAL BREEDING PROGRAMS

Root QTL show great potential for marker assisted selection (MAS) when root traits chosen contribute significantly to drought tolerance in the target environment. The selected root morphology or function for use in MAS can vary greatly depending on the targeted environment and the ultimate goal of the researcher (Blum, 2011). Many of the reported markers and QTL for root traits have proven to be confounded by inadequate root phenotyping, inconsistent contribution across populations and environments, or the minor contributions made by the QTL to the variation in the trait of interest (Collins et al., 2008; Blum, 2011). QTL that have been identified in greenhouse or lab conditions must be validated under field conditions and should ultimately relate to improvements in productivity before use in a MAS program. For these reasons, there have been very few reports of the use of MAS for quantitative traits such as root characters in plant breeding programs. One successful example of a cultivar developed through MAS for root traits is the rice line “Birsa Vikas Dhan 111,” which was selected for a larger root system (Steele et al., 2006). The backcrossing selection scheme used in breeding the rice line targeted five donor-parent chromosomal regions, four relating to root traits and one to end-use quality. In addition, multiple markers were selected for maintenance of the recurrent parent background. Work conducted by Mace et al. (2012) on nodal root angle QTL in sorghum (*Sorghum bicolor*) is an example of relating root QTL to grain yield. These authors tested a subset of the QTL mapping population in yield trials where they identified an association between grain yield and three out of the four lab-identified nodal root angle QTL.

Utilization of molecular markers that improve productivity under drought has been, and will continue to be, a daunting challenge in crop improvement. Because root variation is difficult to phenotype in a breeding population of hundreds of genotypes, MAS offers breeders the option to select for favorable combinations of traits both above and below ground. However, in order for MAS to be successfully adopted by plant breeding programs, either molecular markers must be identified that are in strong linkage disequilibrium with the QTL for desired root traits or the gene itself must be identified. The major obstacle for the use of MAS then becomes accurate phenotyping that can lead to greater accuracy of QTL locations in high density molecular maps (Francia et al., 2005).

### RESOURCES FOR GENETIC DIVERSITY

A reduction in diversity of crop species due to domestication or subsequent selection has been described as a genetic bottleneck that may have contributed to a loss in useful alleles (Tanksley and McCouch, 1997). Root traits are no exception as the importance of developing improved root systems has often been overlooked (Herder et al., 2010). In recent years, improvements in genotyping procedures and knowledge of root architecture have made significant advances due to research in model species such as *Arabidopsis* (Benfey et al., 2010), rice (Hochholdinger and Tuberosa, 2009), and purple false brome (*Brachypodium distachyon*; Draper et al., 2001). The use of model systems offers several advantages. First, comparative mapping of QTL identified to the locations of those QTL in related species is a starting point for candidate gene identification and potential future use in MAS programs (Edwards and Batley, 2010). Second, the use of cloned genes from model systems may be used in altering trait expression in the species of interest through transgenic breeding approaches (Keller et al., 2007; Blum, 2011).

With a better understanding of root traits and their genetics, improvements in root systems can be made by utilizing the diversity currently found within modern cultivated germplasm (Blum, 2011). For example, a comparable amount of unexploited genetic variation contributing to stress tolerance can be found in modern cultivars as in landraces (primitive varieties) of wheat (Trethowan and Mujeeb-Kazi, 2008). Moreover, alleles contributing to more extensive root growth and distribution may be present in cultivated varieties of rice rather than in wild species if observations from container-grown plants hold (Liu et al., 2004). Introgression of alleles from modern varieties reduces the negative effects of linkage drag from the use of wild species and landraces (Hübner et al., 2013). Nonetheless, landrace varieties for certain species may also show potential for introgression of genetic diversity into modern varieties. Not all landrace varieties or wild accessions should be expected to show abiotic stress tolerance, but successful use of this approach can be seen in crops such as barley (Ceccarelli and Grando, 1991), wheat (Trethowan and Mujeeb-Kazi, 2008), and pearl millet (Yadav, 2008).

## PATTERNS OF ROOT TRAITS AND RESPONSES OBSERVED FROM SCREENING STUDIES – CASE STUDIES IN LESQUERELLA AND RICE

We will summarize advances made in two contrasting crops, lesquerella and rice. In the first case, screening studies are just beginning on the emerging oilseed crop, lesquerella, for which improvement is an initiative of the U.S. Department of Agriculture. In the second case, screening studies are quite advanced on rice, a dietary staple for many people. Root trait screening in wheat, which is also advanced, is not reviewed here because it is well covered in recent reviews (Richards, 2006; Wasson et al., 2012).

### LESQUERELLA

Lesquerella [*Physaria fendleri* (A. Gray) O’Kane & Al-Shehbaz] is a C_3_ dicot and member of the Brassicaceae family. Herbaceous lesquerella plants have yellow flowers and are commonly found on calcareous soil in hot arid environments in the U.S. Southwest (Rollins and Shaw, 1973; Al-Shehbaz and O’Kane, 2002). Since the early 1980s, lesquerella has been domesticated and bred as a new oilseed crop in the U.S. because its unique seed oil contain hydroxy fatty acids that have practical applications in industrial manufacturing and added utility as an additive to biofuels (Hinman, 1984; Thompson and Dierig, 1994; Isbell and Cermak, 2002). The target environments for growing lesquerella are Arizona, New Mexico, and Texas where it can be grown as a winter annual crop. Water management involves keeping the field moist until seedling emergence and ensuring that the plants receive about 635–762 mm of water during the growing season for optimal yields, similar to winter wheat (Wang et al., 2010).

Lesquerella has a well-developed tap root system (Rollins, 1981) which has not been well characterized to date. Past screening studies were not designed to focus solely on roots but were conducted simultaneously with observations on the crop for other agronomic traits, seed yield and total biomass in particular.

Although large root biomass allocation has been associated with drought tolerance in many plant species, this characteristic allocation pattern is also associated with a perennial growth form (Chapin et al., 1993). The perennial *Physaria* species *P. mendocina* and *P. pinetorum* were found to generally accumulate greater root biomass than annual forms (González-Paleo and Ravetta, 2011). However, seed yield (biomass) of *P. mendocina* was similar to that of annual *P. fendleri* when both species were grown under water limited conditions (Ploschuk et al., 2001, 2005).

Planting density and stature influence lesquerella’s taproot length, which was reported to grow deeper with increased planting density (110 mm at 250,000 plants ha^-1^ and 180 mm at 750,000 plants ha^-1^; Brahim et al., 1998). Brahim et al. (1998) suggested that deeper rooting in response to increased planting density enabled deeper water and nutrient acquisition to ameliorate increased interplant competition for soil resources.

Various environmental factors affect *Physaria* root traits. In the perennial species *P. ludoviciana*, total root length and branching was greater when plants were grown in growth chambers under medium light intensity (584 μmol m^-2^ s^-1^) than low light intensity (174 μmol m^-2^ s^-1^), which matches its seasonal cycle (Grant, 2009). In *P. fendleri*, genotypes respond differently to growth temperatures, with a number of accessions producing larger root systems under higher temperatures (Cruz et al., 2012). Although individual environmental factors were found to affect root traits, interactions among environmental factors affecting root systems have not been fully studied in *Physaria*. In maize, for example, plant performance under water-limited plus high temperature conditions was different than that under water-limited conditions alone (Cairns et al., 2013).

Characterization of lesquerella germplasm accessions in the U.S. National Plant Germplasm System is underway to determine non-adaptive (constitutive) root traits correlated with increased productivity under drought conditions in improved cultivars of other crops. The methodologies being utilized involve analyzing roots from seedlings in growth pouches, as well as samples grown in the greenhouse and in experimental fields in Maricopa, AZ, USA. Preliminary results of phenotypic evaluation indicate that the relative root size of young plants is maintained through crop maturity (Cruz et al., unpublished). More focused analysis of lesquerella root responses to varying environmental and cultural management conditions will determine if lesquerella has unique responses to abiotic stress compared to major commodity crops, potentially associated with the origin of lesquerella from hot and arid environments.

### RICE

Rice, a monocot and a member of the Poaceae (or Gramineae) family, grows in a wide range of environments and cropping systems have been adapted for deep-water, rain-fed lowland, upland, and irrigated conditions (De Datta, 1981). The genetic and genomic resources on rice are tremendous with the species studied as a model organism for monocot crops, similar to *Arabidopsis* for dicots as mentioned earlier (Rensink and Buell, 2004; Coudert et al., 2010). The drought environments of rice are classified based on the duration of the wet season, as well as the severity of water stress at different growth stages (e.g., early in the season during planting, at the tillering to flowering stages, which is typically intermittent, and during the late season from flowering to grain filling; Fukai and Cooper, 1995).

Studies have been conducted on the influence of rice roots on crop productivity. Research is already in advanced stages compared to lesquerella and most other crops (Henry, 2012). Rice has a well-described fibrous root system characteristic of monocots and exhibits seminal, nodal, and lateral roots which have been subjected to substantial morphometric, anatomical, and genetic studies (Yoshida and Hasegawa, 1981; Morita and Nemoto, 1995; Rebouillat et al., 2009). Regardless of the ecosystem where rice breeding is aimed, researchers look toward understanding the role of roots for improving nutrient and water acquisition and increasing grain yield.

Tropical japonica types have been known to have fewer tillers and deeper root systems than other rice ecotypes (i.e., indica, aus, rayada; Lafitte et al., 2006). There are significant differences reported in root thickness, depth, and root mass among rice cultivars and there is documented genetic variation for root morphological traits in response to drought (Kondo et al., 2003; Gowda et al., 2011). However, this variation and how it influences the crop’s root function for water uptake under drought remains to be fully understood (Gowda et al., 2011). Breeding activities toward a rice plant ideotype and direct selection for yield under drought are underway, supported by physiological studies on rice root function (e.g., root hydraulic conductance, anatomy, and aquaporin expression; Henry, 2012). So far broad examinations of traits show that traits do not appear inherently different between upland and lowland types. Indica types (mostly lowland) have thinner, shallow roots while aus types (often grown upland) exhibit intermediate diameter with length similar to japonicas (which include upland Asian and temperate cultivars; Henry et al., 2012).

Environmental factors and water management practices strongly affect rice root systems. Intermittent irrigation was found to positively affect RLD and total root mass (Shi et al., 2002; Mishra, 2012; Cruz et al., unpublished). Additionally, root size is highly correlated to available growing space, root impedance, and type of existing competitor plants (Fang et al., 2013). Upland rice develops a longer root system compared to lowland counterparts due to environmental factors in these ecosystems (Yong et al., 2007; Fageria, 2013). Well-drained soils in upland areas do not restrict water movement and allow better oxygen diffusion to favor rice root elongation (Yoshida and Hasegawa, 1981; Fageria et al., 2003). Anaerobic flooded field conditions of lowland ecosystems on the other hand can impair root elongation as well as the formation of root hairs (Kawata and Ishihara, 1959; Kawata et al., 1964).

The structure and development of rice root systems largely determines crop functioning under drought (Morita and Nemoto, 1995). Rice improvement programs have determined that deep rooting is a target trait (Gowda et al., 2011). Among upland varieties, cultivars with thicker coarse roots that create an overall deeper root system are generally viewed as desirable under drought conditions along with varieties that have greater RLD in deeper soil layers (Passioura, 1982; Kondo et al., 1999; Steele et al., 2006). Studies of lowland varieties are likewise ongoing to screen for thicker coarse roots to penetrate hardpan soil layers (Gregorio and Cabuslay, 2004; Allah et al., 2010a; Gowda et al., 2011). Greater fine root (lateral) growth has also been found to increase water uptake and rice yield under drought but the mechanism is being further investigated (Henry, 2012).

Various screening methods used to identify root traits associated with drought tolerance in rice germplasm. Root dry mass and length, commonly assessed by direct evaluation, is a good predictor of yield in rice (Beyrouty, 2002; Fageria and Moreira, 2011). Root pulling resistance is also a trait that is highly correlated with root length, thickness, branching number, and dry mass in rice (Price et al., 1989). Root pulling resistance is recommended as an indirect screen to select genotypes that achieve drought tolerance *via* producing a large root system (Ekanayake et al., 1985; Lafitte et al., 2006). Additionally, researchers used the number of root xylem vessels to gage drought resistance of rice lines (Allah et al., 2010b). However, there is substantial variation in the distribution of xylem vessels across rice roots with lowland rice generally reported to have fewer root xylem vessels than upland rice at the middle and tip sections of the root (Bashar, 1990).

Rice root traits are currently characterized using greenhouse container methods or field sampling techniques, both high-throughput but labor intensive (Gregorio and Cabuslay, 2004; Shashidhar et al., 2012; Cruz and Dierig, unpublished). Root imaging technologies are allowing a closer look at the dynamic nature of rice root system architecture and these present opportunities to fast track understanding the genetic control of root traits, specifically lateral branch formation. Non-invasive imaging techniques provide important insight on spatial distribution of rice roots and might allow the identification of genetic control over rice root system architecture. However, most imaging studies require plants to be grown in artificial media. Further testing is needed to determine if rice root systems traits observed in artificial media are found under actual field conditions (Clark et al., 2011; Feng et al., 2012). Several mutant lines of rice are being used in studies of the molecular control of lateral root branching (Smith and De Smet, 2012). Molecular studies are also examining genes and signaling pathways that control morphological response to drought (Fukao and Xiong, 2013). Ultimately, further advances in phenotyping methodologies and field validation are needed to link traits identified in these studies to drought resistant in rice varieties (Virmani and Ilyas-Ahmed, 2007).

Several rice germplasm collections in genetic resources centers have been screened for root traits associated with drought tolerance and promising accessions have been identified as useful in breeding programs (Chang and Loresto, 1986; Henry et al., 2011). Examples of germplasm selected for drought related studies include those with (1) high levels of drought tolerance with deep and thicker root systems (e.g., OS4, Salumpikit, Azucena), (2) moderate drought tolerance and early maturity (e.g., Dular, Black Gora, Bala), and (3) improved drought tolerance and ability to produce new tillers after soil water replenishment (e.g., IR43, IET1444, UPLR-5; Virmani and Ilyas-Ahmed, 2007). In addition to cultivated forms, root systems of wild rice germplasm have been characterized for contributions to drought resistance with *O. longistaminata* and *O. rufipogon* identified as potential sources of novel alleles for drought tolerance (Liu et al., 2004). Superior performance under water stressed conditions in the greenhouse was correlated with the production of greater root system length and greater root to shoot ratios when exposed to drought conditions (Feng et al., 2012).

A small set of rice root QTL have been identified and were found to result in increased root penetration, thickness, nodal root apex stiffness and length when introgressed into rice lines (Steele et al., 2006; Clark et al., 2008). These QTL contribute positively in different test environments and the different combinations of the QTL all exhibited advantages in water uptake making them important in crop improvement activities in rice (MacMillan et al., 2006; de Dorlodot et al., 2007). Field testing of upland rice in India with four introgressed QTL were found to produce plants with longer root lengths and a yield advantage of 1 t ha^-1^ compared to controls (Steele et al., 2006). Additionally, transgenic rice plants with increased root diameter, developed by overexpressing *OsNAC5*, were found to increase yield by 9–26% (Jeong et al., 2013).

These practical applications from decades of root research in rice and in other model systems will enable further understanding of important traits that might influence crop yield and productivity under abiotic stress and ensure gains toward global food security.

## CONCLUSION

There is maturing promise for breeding plants with root traits to enhance productivity under water deficit. Although much is known about root traits and functioning, there is a need for better understanding of traits in the context of plant strategies for growth under water deficits. A better understanding of tradeoffs in root traits is also needed to guide breeding efforts. Although breeding different crops for specific forms of drought needs to be carefully considered with particulars of different systems in mind, certain generalities for root traits may hold. Smaller diameter roots, greater SRL, and increased root hair density or length should improve plant acquisition of water under water scarcity and reduce plant carbon investment required for that acquisition. Additionally, crop hydraulic functioning under water scarcity may be improved through increased capacity for nocturnal refilling of embolized xylem and changes in inter-vessel pit anatomy to reduce cavitation, which may not carry negative repercussions under well-watered conditions. The ability of plants to access water from deep depths in the soil profile has been documented and found to benefit crop productivity under water scarcity. Deep water acquisition, however, does not necessarily fully ameliorate crop water requirements during hot dry conditions, even when deep soil water is available (Sun et al., 2011), suggesting that more information is needed on root–shoot interactions governing hydraulic conductance, especially under high temperatures and vapor pressure deficits (e.g., Yang et al., 2012b). Basic information on seasonal growth patterns, essential to understand effective plant capacity for and control over root hydraulic conductance with plant development over the season, especially for woody plants, is frequently missing or incorrectly assumed and is needed to guide breeding efforts (Comas et al., 2005; Eissenstat et al., 2006). While water uptake capacity declines with root aging and exposure to dry soil (Lo Gullo et al., 1998), it is unclear if new root production is required to maintain root hydraulic conductivity or if enhanced aquaporin activity can ameliorate uptake capacity. Abundant progress has been made in understanding root traits and functioning in plant water acquisition with several root QTL identified. There continue to be promising prospects for increasing communication between plant ecophysiologists, geneticists, and breeders to learn more about root traits that have the potential to improve plant productivity under drought and put this understanding into practice to improve the performance of crops under water shortages.

## Conflict of Interest Statement

The authors declare that the research was conducted in the absence of any commercial or financial relationships that could be construed as a potential conflict of interest.

## AUTHOR CONTRIBUTIONS

LHC wrote sections pertaining to root traits, growth, and distribution. SRB wrote sections on genetic analyses of root traits with assistance from PFB. VMVC wrote sections on lesquerella and rice trait screening with assistance from DAD.
